# Dysregulation of angiogenesis-specific signalling in adult testis results in xenograft degeneration

**DOI:** 10.1038/s41598-017-02604-4

**Published:** 2017-06-01

**Authors:** Lalitha Devi, Lavanya Pothana, Sandeep Goel

**Affiliations:** Laboratory for the Conservation of Endangered Species, Centre for Cellular and Molecular Biology, Council for Scientific and Industrial Research, Uppal Road, Hyderabad, 500 007 India

## Abstract

Ectopic xenografting of testis is a feasible option for preservation of male fertility and angiogenesis plays a pivotal role in xenograft survival and functionality. When compared to immature testis, the adult testis is unable to establish functional xenografts due to potentially lower efficiency to induce angiogenesis. The precise molecular mechanism, however, remains elusive. In the present study, we compared adult and immature testis xenografts for survival, maturation and germ cell differentiation. Further, we evaluated differential expression of angiogenesis signalling-specific proteins in adult and immature testis and their xenografts. Results showed that adult testis xenografts degenerated whereas immature testis xenografts survived and established spermatogenesis with the production of haploid germ cells. Protein expression analysis demonstrated that immature testis xenografts were able to establish angiogenesis either through eNOS activation via VEGF and PI3K/AKT or through EGFR-mediated STAT3 pathway. The role of ERK/MAPK pathway in xenograft angiogenesis was ruled out. The absence or reduced expression of angiogenesis-specific proteins in adult testis and its xenografts possibly resulted in poor angiogenesis and in their subsequent degeneration. This study provides insight into angiogenesis mechanism that can be utilized to augment testis xenografting efficiency.

## Introduction

Ectopic testis tissue xenografting is a feasible technique for studying spermatogenesis and testicular maturation. This technique has been used for the production of mature gametes by grafting small pieces of testis tissue under the dorsal skin of immunodeficient mice recipients^1^. Testis tissue xenografting allows for modulation/modification of spermatogenesis by manipulation of recipient mice environment. Several factors such as the size of the tissue, temperature at the grafting site and period of hypoxia play a vital role in the success of xenografting. The most important event that ensures survival of grafted testis tissue is the induction of angiogenesis. Interestingly, testis from differently aged donors has different potential for development when grafted onto recipient mice^2^. Till date, only testis from sexually immature hosts have resulted in successful progression of xenografts to complete spermatogenesis and emerged as successful models for studying testicular development *in vivo*. Fresh immature testis tissues from several species such as monkeys^3, 4^, bulls^5, 6^, buffalo^7^, goats^1^, pigs^1^, dogs^8^, cats^9^, hamsters^10^, rabbits^11^ and mice^1, 11^ have shown complete spermatogenesis following xenografting onto mice recipients.

Despite poor outcome of ectopic xenografting of adult testis tissue, it could be a feasible option to maintain spermatogenesis in testis that is rendered azoospermic by disease or cancer treatment^12^ or in species with seasonal reproductive patterns outside of mating season^13^. Xenografting of adult testis can also be used for studying the mechanism that causes impairment of spermatogenesis^14^. However it has been observed that in the xenografted human adult testis, germ cells fail to differentiate^15, 16^ and spermatogenesis was arrested at meiosis in grafted horse^17^, dog^8^ and in the photo-regressed hamster^18^. Similarly, testes from mature adult donor goat, pig and cattle have not supported germ cell differentiation in xenograft^18^. Complete spermatogenesis leading to formation of elongated spermatids in xenografted testis from sexually mature species has not been reported so far. The failure of sexually mature testis to establish functional xenografts is attributed to their greater sensitivity to ischemia when compared with immature tissue. Testis from sexually mature animals are potentially less effective in inducing angiogenesis^19^. The angiogenesis in grafted tissues is regulated by the expression of angiogenesis signalling-specific proteins in the donor testis. Expression of several angiogenesis signalling-specific transcripts have been reported in immature bovine testis however, their role in angiogenesis needs to be determined^2^. There is no report so far which specifically identifies differentially expressed key angiogenesis signalling-specific proteins in immature and adult testis xenografts.

Angiogenesis or the formation of new blood vessels involves a complex and dynamic interaction between endothelial cells and the corresponding extracellular environment^20^. The survival and function of testis xenografts majorly depend on the rapid formation of a stable microvasculature between the host and the grafted tissue^18, 21^. The grafted tissue must establish an efficient blood vessel connection to the host’s circulatory system rapidly to facilitate the supply of oxygen and nutrients^22^. A successful xenograft should have adequate angiogenesis for functional Sertoli cells, which in turn are necessary for functional germ cells^2^. It has been reported that host contributes towards the formation of major connecting blood vessels associated with the graft and host^22^. However, cell signalling in testis tissue xenografts that induces angiogenesis is not yet known.

The objective of the present study is to evaluate the differentially expressed key-angiogenesis signalling-specific proteins in adult and immature testis xenografts to understand their role in angiogenesis.

## Results and Discussion

### Weight of seminal vesicle of recipients, xenograft recovery and histological analysis

At 8 wk post grafting, xenografts and seminal vesicles were recovered from recipients grafted with adult testis tissues or immature rat testes and weighed (Table 1). The weight of seminal vesicle is a reliable indicator of circulating bioactive testosterone and has been used in several studies^1, 7, 17, 23–25^. The average weight of seminal vesicles recovered from recipients grafted with adult testis tissues was significantly lower than from those grafted with immature testis and non-grafted intact control mice (131 ± 3.1 mg; P < 0.05). The inability of xenografts from adult testis to produce sufficient testosterone could be due to lack of mature Leydig cells. These results contradict previous findings where restoration of seminal vesicle weight in mice^26, 27^ and, serum testosterone level in rats^28^ following adult testis autografting has been reported. This inconsistency could be due to failure or delay in the establishment of angiogenesis in xenografted adult testis leading to loss of Leydig cells^29^. The recovery of seminal vesicle weight in recipients grafted with immature testis suggests that not only the spermatogenic but also the steroidogenic function of the immature rat testis was restored in the xenografts. Induction and maintenance of spermatogenesis require a continuous and controlled interaction of several hormones in the hypothalamic–pituitary–testis axis^30^. This indicates that a synchronized hormonal interaction was established between the recipient’s hypothalamus and pituitary and the xenografted immature rat testis, which induced spermatogenesis in the xenografted tissue. Thus, suggesting that Sertoli cells and Leydig cells present in donor tissue were able to respond to mouse gonadotrophin stimulation.

**Table 1:** Experimental graft data for testis of immature and adult rat donors xenografted and collected 8 wk post grafting.

Tissue type	Number of mice grafted and analyzed	Seminal vesicle weight (mg)	Average graft weight (mg)	Number of grafts recovered^a^ (%)	Number of healthy grafts^b^ (%)	Number of grafts with meiotic germ cells^c^ (%)
Adult rat testis tissue	10	31 ± 2.1*	42 ± 3.8*	82.5 (33/40)*	0 (0/33)*	0 (0/0)*
Young rat testis tissue	10	128 ± 4.7	132 ± 6.2	100 (40/40)	75 (30/40)	83.3 (25/30)

^a^Total xenografts removed divided by the total number of xenografts grafted. ^b^Percentage of recovered xenografts with intact seminiferous tubules. ^c^Percentage of recovered xenografts with pachytene-stage spermatocytes. *Significantly different within the column (P < 0.05).

Average graft weight and total graft recovery were significantly lower from recipients grafted with adult rat testis tissue than in recipients grafted with immature rat testis (P < 0.05). The lower graft recovery from recipients grafted with adult rat testis tissue can be attributed to complete degeneration of xenografts and lower xenograft weight to progressive disintegration of the testicular architecture and extensive degeneration of seminiferous tubules.

**Figure 1:**
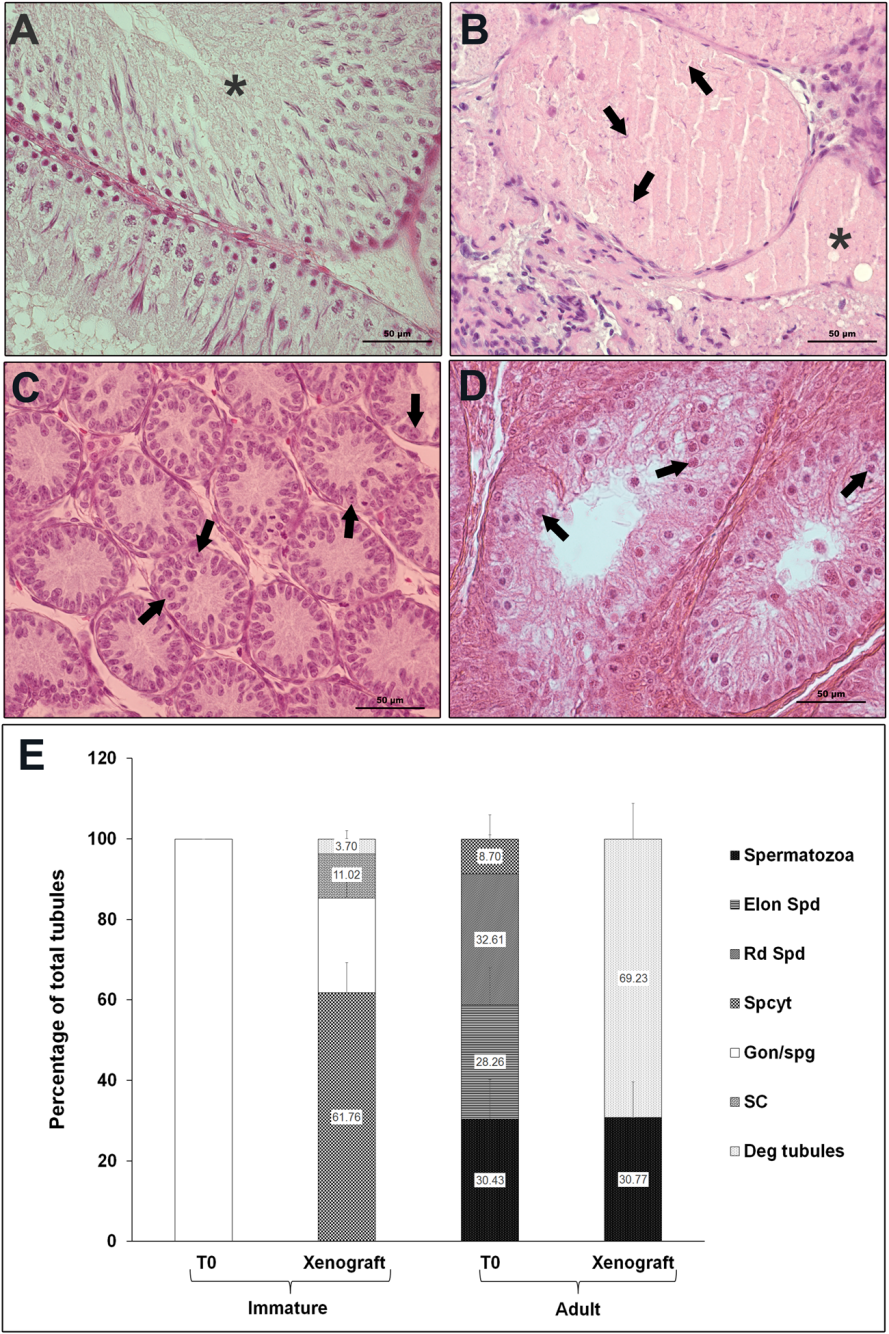
Histological examination and quantitative assessment of seminiferous tubules for the most advanced germ cell type. (**A**) In adult donor tissue from 10-wk-old rat (T0). Seminiferous tubule with normal spermatogenesis is indicated by an asterisk. (**B**) In adult rat testis xenograft at 8-wk-post grafting. Degenerated seminiferous tubules are indicated by asterisks and arrows indicate sperm. (**C**) In immature donor tissue from 6-day-old rat (T0). Note that the most advanced germ cells at this age were gonocytes/spermatogonia (arrows). (**D**) In immature rat testis xenograft at 8-wk -post grafting. Note that the most advanced germ cells identified in the xenografts were pachytene-stage spermatocytes (arrows). (**E**) Percentage of seminiferous tubules with the most advanced germ cell type. Deg tubules, degenerated tubules; SC, Sertoli cell only; Gon/spg, gonocytes or spermatogonia; Spcyt, pachytene spermatocytes; Rd Spd, round spermatid; Elon Spd, elongated spermatid; Spermatozoa, spermatozoa. Data are presented as mean ± SEM. Bars with different letters are significantly different at P < 0.05. Scale bar = 50 µm.

Histological evaluation of 10-wk-old donor rat testis showed intact seminiferous tubules with normal spermatogenesis whereas 6-day-old rat donor testis showed seminiferous tubules with spermatogonia as the most advanced germ cell in 100% tubules (Fig. 1A,C and E). Xenografts from adult rats showed extensive degeneration of the tubules (Fig. 1B) with few sperm in 30.77 ± 8.8% tubules (Fig. 1E). These findings are consistent with those from previous studies, which reported that adult testis tissue underwent regression or extensive sclerosis following ectopic xenografting^10, 16, 18^. Moreover, the adult testis tissue xenografts are also highly sensitive to ischaemia^10^ and have a high demand for oxygen because of ongoing spermatogenesis^22^. The survival of sperm cells in otherwise degenerated tubules is interesting. It is likely that spermatozoa being terminally differentiated cells are rather resistant to degeneration and are able to survive in degenerated tubules. We had observed similar findings in autografted adult mice testis^27^ and another report also confirmed similar finding^16^. Testis from immature donors could establish spermatogenesis following ectopic xenografting and meiosis was induced as indicated by the presence of pachytene-stage spermatocytes as the most advanced germ cells (Fig. 1D and E). These results are consistent with a previous report in which, xenografted rat testis could not complete spermatogenesis following an 8 wk grafting period^22^. Although 8-wk-old rat testis showed complete spermatogenesis *in situ* (data on file), its xenograft was unable to do so. This could be due to initial ischemia before the blood supply to the grafts is established leading to a delay in initiation of spermatogenesis. Whether a longer grafting period is required for spermatogenesis to get completed in xenografted immature rat testis needs to be evaluated.

### PCNA immunostaining

**Figure 2:**
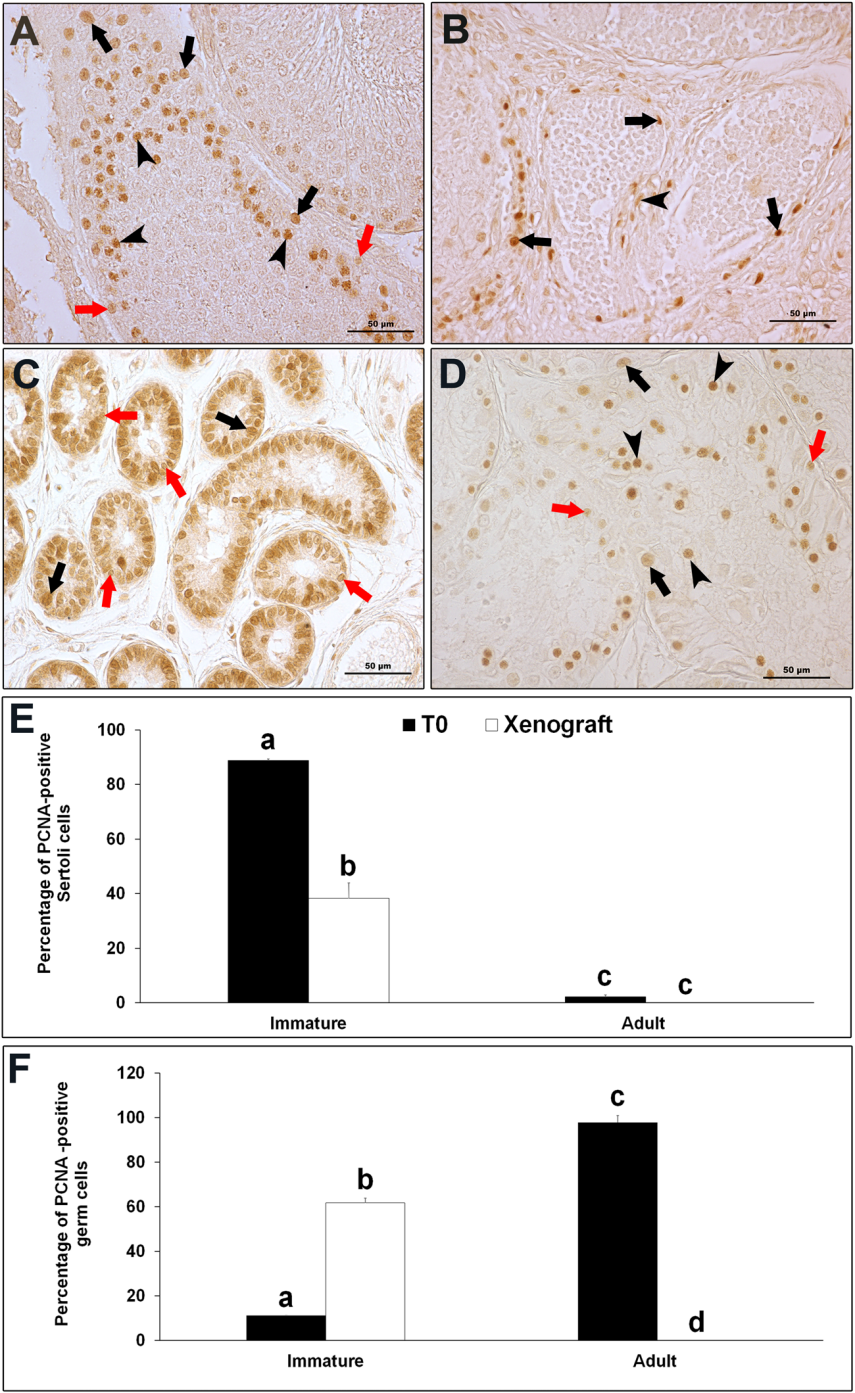
Proliferating cell nuclear antigen (PCNA) immunostaining (**A**) In donor tissue from 10-wk-old rat (T0). Black arrows indicate PCNA-positive spermatogonia, red arrows indicate PCNA-positive Sertoli cells and, arrow heads indicate PCNA-positive spermatocytes. (**B**) In adult rat testis xenograft at 8-wk-post grafting. Note that no PCNA-stained cells are present inside the seminiferous tubules. Although, PCNA-stained cells in the peri-tubular region (arrows) and in interstitial space (arrow head) were observed. Expression of PCNA protein in (**C**) In immature donor tissue from 6-day-old rat (T0) and, (**D**) Immature rat testis xenograft at 8-wk -post grafting. Black arrows indicate PCNA-positive spermatogonia, red arrows indicate PCNA-positive Sertoli cells. In (**D**), arrow heads indicate PCNA-positive spermatocytes. (**E**) Percentage of PCNA- positive Sertoli cells and (**F**) Percentage of PCNA- positive germ cells in donor and grafted testes. Data are presented as mean ± SEM. Bars with different letters are significantly different at P < 0.05. Scale bar = 50 µm.

PCNA immunostaining was localized in the nuclei of all the dividing cells. In 10-wk-old donor rat testis, a strong PCNA staining was evident in proliferating spermatogonia, spermatocytes and in a few Sertoli cells (as indicated by their nuclear morphology and location in seminiferous tubule) in the seminiferous tubules (Fig. 2A). In 6-day-old immature donor testis, strong PCNA staining was evident in Sertoli cells and germ cells (Fig. 2C). The percentage of PCNA-positive Sertoli cells was significantly higher in testis of immature donors than in testis of adult donors (Fig. 2E; P < 0.05). On the contrary, the number of PCNA-positive germ cells was significantly higher in testis of adult donor than that in testis of immature donor (Fig. 2F; P < 0.05). Grafts from immature donor collected at 8 wk showed PCNA-positive pachytene-stage spermatocytes and spermatogonia (Fig. 2D). However, few Sertoli cells were also stained by PCNA antibody in these xenografts. Number of PCNA-positive Sertoli and germ cells were quantified to assess the proliferation activity and maturation status of the xenografts (Fig. 2E and F). There was a significant increase in the number of PCNA-positive germ cells and a significant reduction in the number of PCNA-positive Sertoli cells in immature testis xenografts (P < 0.05). These findings suggest maturation of xenografts in recipient mice as reported previously^7, 31^. Sertoli cell proliferation, maturation and establishment of spermatogenesis further confirmed that a synchronized hormonal interaction was indeed established between the recipient’s hypothalamus and pituitary and the xenografted immature rat testis. However, a significantly lower number of PCNA-positive Sertoli cells in grafts than that in the adult testis implied incomplete maturation. This delayed maturation of grafts could be a possible reason for incomplete spermatogenesis in the grafted testis. In grafts from adult donor, no PCNA-stained cells were evident in the tubules (Fig. 2B). These results further confirmed degeneration of grafts from adult testis. Interestingly, PCNA-stained cells were observed in the peritubular region and interstitial space in adult testis grafts. These PCNA-stained cells possibly represent stem or foetal Leydig cells as reported previously^27^.

### Expression of angiogenesis signalling-specific proteins in xenograft

**Figure 3:**
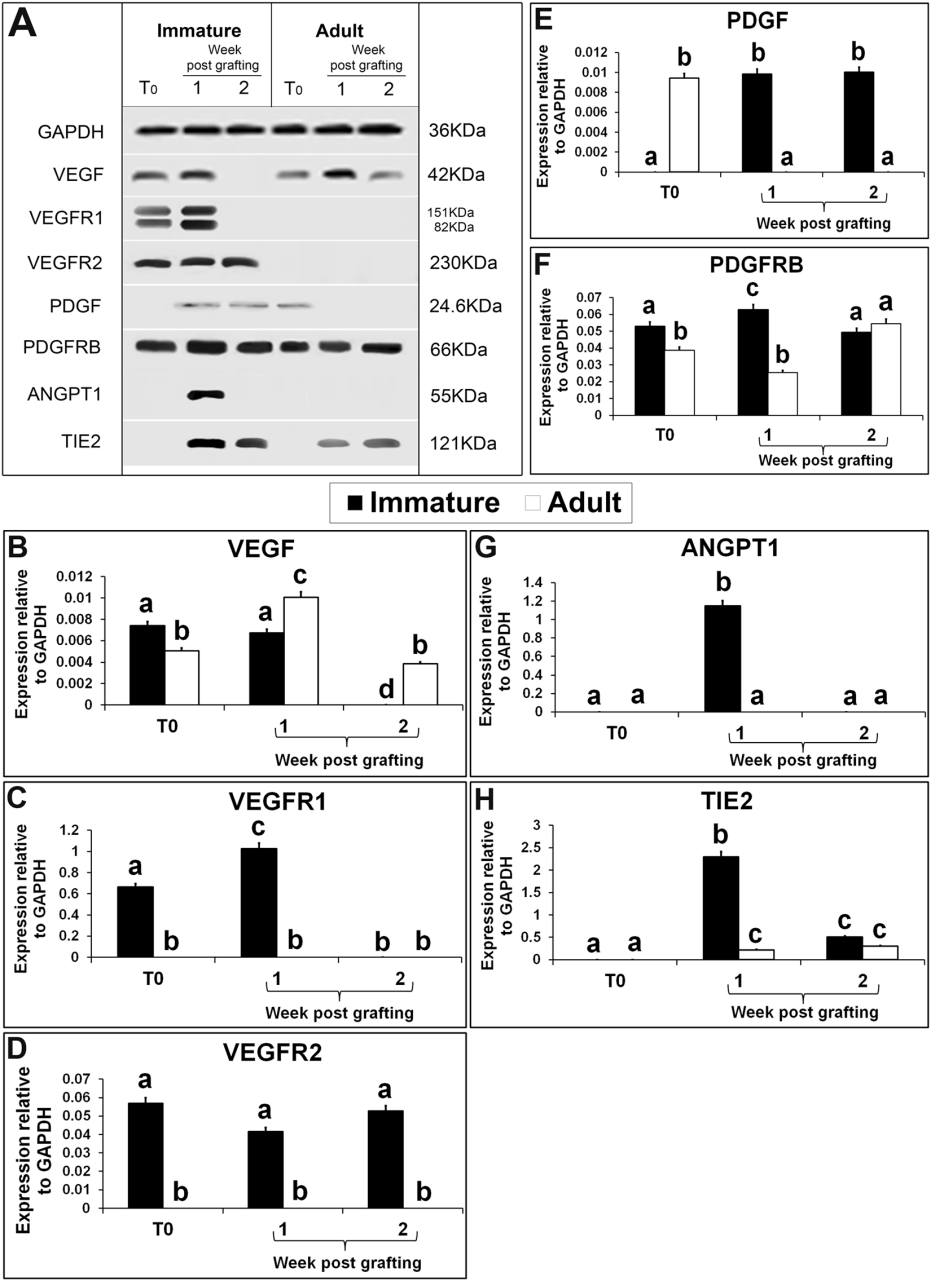
Western blot analysis of xenografted immature and adult testes at 1-and 2-wk-post grafting for expression of growth factors and their receptor-specific proteins. Protein expression in 6-day-old and 10-wk-old donor testes before grafting (T0) are presented as starting material. Representative blot (**A**) and densitometry analysis of (**B**) VEGF, (**C**) VEGFR1, (**D**) VEGFR2, (**E**) PDGF, (**F**) PDGFRB, (**G**) ANGPT1, and (**H**) TIE2 protein. Y-axis represents intensity of bands relative to GAPDH. Data are presented as mean ± SEM. Bars with different letters are significantly different at P < 0.05.

Xenografts from recipients grafted with immature and adult testis were analysed for the expression of various angiogenesis signalling-specific proteins. Since angiogenesis is completed in xenografts 2 wk post grafting and there is no temporal change in the extent of angiogenesis thereafter^32^ hence, xenografts were analysed 1 and 2 wk post grafting. Growth factors and their receptors play an important role in the initiation of angiogenesis and the generation of new blood vessels critical for graft survival. VEGF is a key regulator of both vasculogenesis and angiogenesis^33^. In the present study, the expression of VEGF protein was present both in testis of immature and adult donors but its expression level was significantly lower in adult testis (Fig. 3A and B; P < 0.05). VEGF expression in the xenografts from immature testis collected at 1 wk was similar to that in immature donor testis (P > 0.05) however; its expression was undetectable at 2 wk (P < 0.05). In xenografts from adult testis, VEGF expression level increased at 1 wk (P < 0.05) and then reached the same level as at baseline at 2 wk (P > 0.05). VEGF is a potent angiogenic factor that stimulates blood vessel development and migration^34^. In a previous report, treatment of immature bovine testis tissues with VEGF has shown to increase graft survival and germ cell differentiation^35^. Interestingly, the expression of VEGF receptors, VEGFR1 and VEGFR2, was present only in immature testis and in their respective xenografts (Fig. 3C and D). Of the three VEGF receptors, only VEGFR1 and VEGFR2 are reported to play a predominant role in angiogenesis^36^. The persistent expression of VEGF and its receptors in immature testis xenografts at 1 wk indicates active angiogenesis and their subsequent loss at 2 wk indicates completion of angiogenesis. Though an elevated expression of VEGF protein was also detected in adult testis xenografts at 1 wk, the likely absence of its receptors may have limited the angiogenic effect of VEGF.

PDGFs are dimeric proteins that exert their functions by binding to and activating PDGF receptors in the cell membrane^37^. PDGF has been implicated in the formation of new capillary blood vessels and in enhancing capillary growth *in vitro* ^38^. In the present study, the expression of PDGF protein was absent in immature testis but a raised expression was observed in their xenografts, collected at both time points (Fig. 3A and E; P < 0.05). These results contradict with that from a previous report in which *Pdgf* mRNA expression was observed in 5-day-old rat testis^39^. This discrepancy could be due to delay in translation of mRNA into protein in the juvenile rat testis. On the contrary, the expression of PDGF protein was present in adult testis but, was absent in their xenografts at both collection time points. In adult testis, PDGF and its receptors appear to have an essential role during spermatogenic cell differentiation^40^. However, the expression of PDGF in immature testis xenografts may be due to increased blood flow and proliferation of cells as reported earlier^41^. Unlike PDGF, the expression of PDGFRB, a PDGF receptor, was present both in immature and adult testes and their respective xenografts collected at either time points (Fig. 3A). The expression level of PDGFRB was significantly higher in immature testis than in adult testis (Fig. 3F; P < 0.05). Similarly, PDGFRB expression level was significantly higher in immature testis xenografts than in adult testis xenografts at 1 wk (P < 0.05). However, the PDGFRB expression level in both immature and adult testis xenografts was similar at 2 wk (P > 0.05). Interestingly, *Pdgfrb* transcript and protein are reported to be undetectable in non-angiogenic endothelial cells^38^. Therefore, expression of PDGF and PDGFRB protein in the immature testis xenograft indicates active angiogenesis. Further, the difference in their expression levels at the two-time points indicates active angiogenesis at 1 wk and its decline by 2 wk. On the other hand, absence of PDGF in adult testis xenografts could have prevented angiogenesis despite presence of PDGFRB.

ANGPT1/TIE2 signalling is believed to regulate both maintenance of vascular quiescence, promotion of angiogenesis^42^ and prevention of clotting^43–45^. The expression of ANGPT1 and its receptor TIE2 was absent in both immature and adult testes, which suggests their limited role in angiogenesis in normal conditions. However, expression of ANG1 and TIE2 proteins was present in xenografts from immature testis collected at 1 wk (Fig. 3C and D; P < 0.05). Interestingly, ANGPT1 expression was absent in immature testis xenografts collected 2 wk post grafting and in adult testis xenografts at both collection time points. The expression level of TIE2 protein in immature testis xenografts decreased significantly at 2 wk (P < 0.05). In adult testis xenografts, weak expression of TIE2 protein was detected at both collection time points but its level was similar (P > 0.05). Elevated expression of ANGPT1/TIE2 in immature testis xenografts at 1 wk suggests their crucial role in angiogenesis. However, absent or significantly lower expression in adult testis xenografts indicates the poor angiogenic potential of these xenografts.

**Figure 4:**
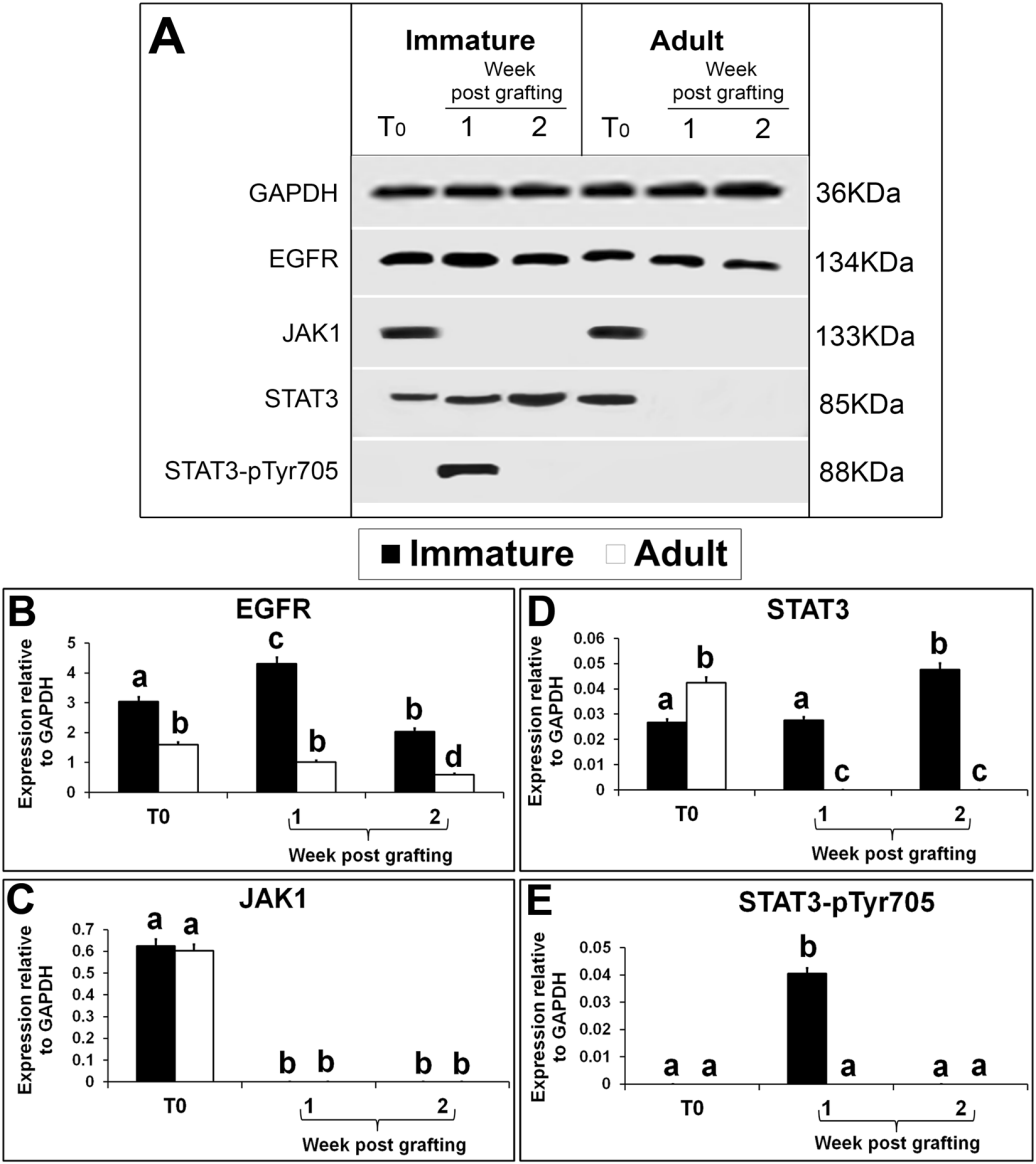
Western blot analysis of xenografted immature and adult testes at 1-and 2-wk-post grafting for expression of EGFR and STAT pathway proteins. Protein expression in 6-day-old and 10-wk- old donor testes before grafting (T0) are presented as starting material. Representative blot (**A**) and densitometry analysis of (**B**) EGFR, (**C**) JAK1, (**D**) STAT3, and (**E**) STAT3 –pSer705 protein. Y-axis represents intensity of bands relative to GAPDH. Data are presented as mean ± SEM. Bars with different letters are significantly different at P < 0.05.

EGFR is a trans-membrane tyrosine kinase receptor which regulates the synthesis and secretion of several angiogenic growth factors including VEGF and bFGF. EGFR is frequently expressed in human carcinomas and supports proliferation and survival of cancer cells^46^. In the present study, the expression of EGFR protein was present both in immature and adult testes but its level was lower in adult testis (Fig. 4B; P < 0.05). EGFR expression level was elevated in immature testis xenografts collected at 1 wk (P < 0.05) but significantly reduced in xenografts collected at 2 wk (P < 0.05). In adult testis xenografts collected at 1 wk, EGFR expression level was similar to that in the donor testis (P > 0.05), but was significantly reduced in xenografts collected at 2 wk (P > 0.05). In either case, EGFR expression level was higher in xenografts from immature testis than in adult testis. Elevated EGFR expression has been reported to enhance proliferation of cancer cells^47^. Similarly, the elevated expression of EGFR protein in immature testis xenografts indicates enhanced proliferation of testicular cells in immature testis xenografts. Higher PCNA stained cells in xenografts of immature testis collected 8 wk post grafting corroborates with this finding. The studies on cancer cells have reported that in response to hypoxia, cells appear to express surface ligands and receptors such as EGFR to initiate cell survival pathways^48^. The activation of EGFR signalling results in activation of several downstream pathways, which may affect transcription or translation of VEGF, including the RAS/ERK/MAPK, PI3K/AKT/mTOR, and STAT3 pathways^49, 50^. Constitutive activation of JAK/STAT pathway occurs commonly in cancer and endothelial cells and contributes to angiogenesis^51^. STAT3 is a critical multifunctional mediator and regulates many aspects of angiogenesis at transcriptional level^52^. The expression of JAK1 protein was present in immature and adult donor testis and its level was similar in both (Fig. 4C; P > 0.05). However, the expression of JAK1 protein was absent in xenografts from immature and adult testis. Similarly, the expression of STAT3 protein was present in immature and adult testes but its level was significantly higher in adult testis (Fig. 4D; P < 0.05). STAT3 protein expression level in immature testis xenografts collected at 1 wk was similar to that in immature testis (P > 0.05) and increased significantly in xenografts collected at 2 wk (P < 0.05). STAT3 expression was absent in adult testis xenografts at both collection time points. STAT3 signalling plays a predominant role in angiogenesis when activated by localization of VEGFR2 onto endothelial cells^53^. Since the expression of JAK1 was absent in xenografts of both immature and adult testis, activation of STAT3 may have occurred through EGFR. To confirm the possibility whether STAT3 is directly involved in angiogenesis in testis xenografts, expression of STAT3-pTyr705, the activated STAT3 protein, was evaluated. Although the expression of STAT3-pTyr705 was absent from immature and adult donor testes (Fig. 4E), STAT3-pTyr705 level was raised in immature testis xenografts collected at 1 wk (P < 0.05). Interestingly, STAT3-pTyr705 expression was undetectable in immature testis xenografts collected at 2 wk and adult testis xenografts at both collection time points. This elevated expression of STAT3-pTyr705 in immature testis xenografts at 1 wk could be a result of rapid phosphorylation of STAT3 protein soon after EGFR activation^54^. These findings suggest a critical role of STAT3 in angiogenesis in grafted testis.

**Figure 5:**
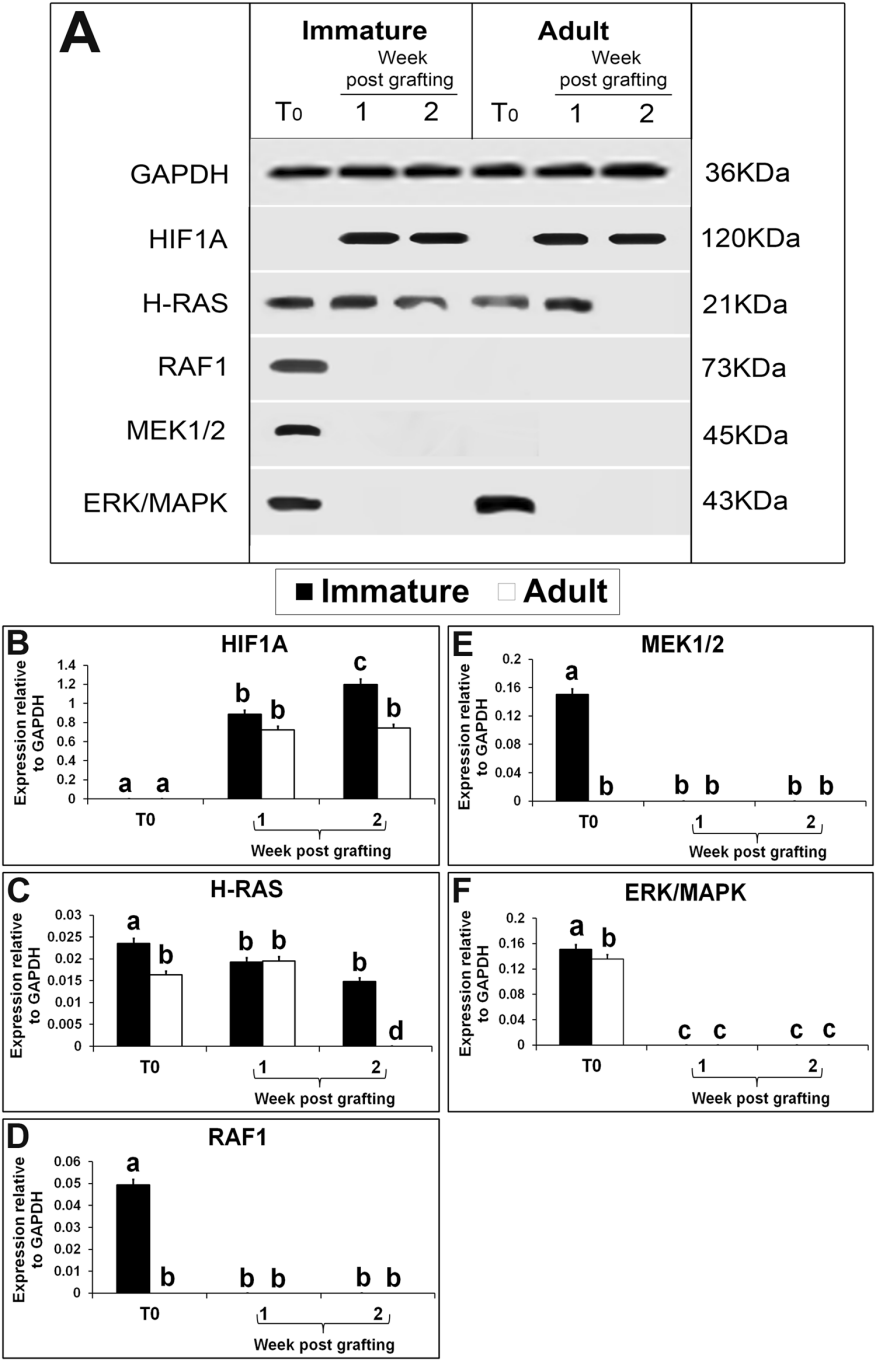
Western blot analysis of xenografted immature and adult testes at 1-and 2-wk-post grafting for expression of hypoxia and ERK/MAPK pathway proteins. Protein expression in 6-day-old and 10-wk- old donor testes before grafting (T0) are presented as starting material. Representative blot (**A**) and densitometry analysis of (**B**) HIF1A, (**C**) H-RAS, (**D**) RAF1, (**E**) MEK1/2, (**F**) ERK/MAPK protein. Y-axis represents intensity of bands relative to GAPDH. Data are presented as mean ± SEM. Bars with different letters are significantly different at P < 0.05.

HIF1A is a hypoxia-related protein that plays a key role in oxygen homeostasis^55^. In the present study, the expression of HIF1A protein was absent from both immature and adult testis (Fig. 5A). Under normoxic conditions, HIF1A subunits are unstable and are rapidly targeted for degradation by proteasome^56, 57^. However, exposure to hypoxia results in a rapid increase of HIF1A protein in many cells^58^. Expression of HIF1A protein in xenografts, irrespective of the maturity status of donor rats, confirmed the presence of hypoxic conditions in grafted testes. HIF1A expression level was not different in xenografts of immature and adult testis collected at 1 wk (Fig. 5B; P > 0.05). However, in the immature testis xenografts collected at 2 wk, significantly higher expression of HIF1A protein was detected than in adult testis xenografts collected at the same time point (P < 0.05). The higher sensitivity of immature testis to hypoxia could be the cause of elevated expression of HIFA1 in immature testis xenografts. This high expression of HIFA1 in immature testis xenografts could have stimulated several downstream angiogenesis-specific signalling pathways. One such crucial signalling pathway is RAS/RAF/ERK/MAPK. The induction of this pathway is either dependent or independent of HIF1A where H-RAS oncogene seems to play a pivotal role^59^. Further, H-RAS is a key downstream effector of the EGFR^60^. H-RAS plays an important role in angiogenesis and is responsible for the induction of angiogenic factors like VEGF, PGF, and ANGPT2 in skin cancer^61^. In response to hypoxia, H-RAS can induce the expression of VEGF that is modulated either by PI3K/AKT or ERK/MAPK pathways^62, 63^. In this study, though the expression of H-RAS was present in both immature and adult testis, its level was significantly higher in immature testis than in adult testis (Fig. 5C; P < 0.05). The expression level of H-RAS was significantly reduced in immature testis xenografts collected at 1 wk versus immature testis (P < 0.05). However, the expression level in immature testis xenografts was same at 1- and 2-wk post grafting (P > 0.05).The expression level of H-RAS protein was similar in adult testis xenograft at 1 wk post grafting and in adult testis (P > 0.05). However, H-RAS expression was undetectable in xenografts 2 wk post grafting. RAF1 is a serine-threonine protein kinase, which initiates an MAPK cascade on activation. The MAPK cascade involves the sequential phosphorylation of the dual-specific MAPK kinases (MAP2K1/MEK1 and MAP2K2/MEK2) and the extracellular signal-regulated kinases (ERK1 and ERK2)^64^. Once activated, ERK1/and 2 phosphorylate several nuclear and cytoplasmic effector proteins involved in diverse cellular responses such as cell proliferation, survival, differentiation, motility, and angiogenesis^65^. Though the expression of RAF1 and MEK1/2 proteins was present in immature testis, it was absent in adult testis and in xenografts of both immature and adult testis (Fig. 5D and E). The expression of ERK/MAPK protein was present both in the immature and adult testis but absent in their respective xenografts (Fig. 3H). Moreover, the expression of activated ERK/MAPK, the MAPKp44/42 protein was also absent in testis of immature and adult donors and their respective xenografts collected at both time points (Fig. 5F). It is evident that H-RAS expression was present in immature and adult testis and their respective xenografts but, the expression of downstream pathway proteins such as RAF1, MEK1/2, ERK/MAPK and MAPKp44/42 was absent in the xenografts. Therefore, the RAS/ERK/MAPK pathway potentially has limited or no role in angiogenesis of xenografted testis.

**Figure 6:**
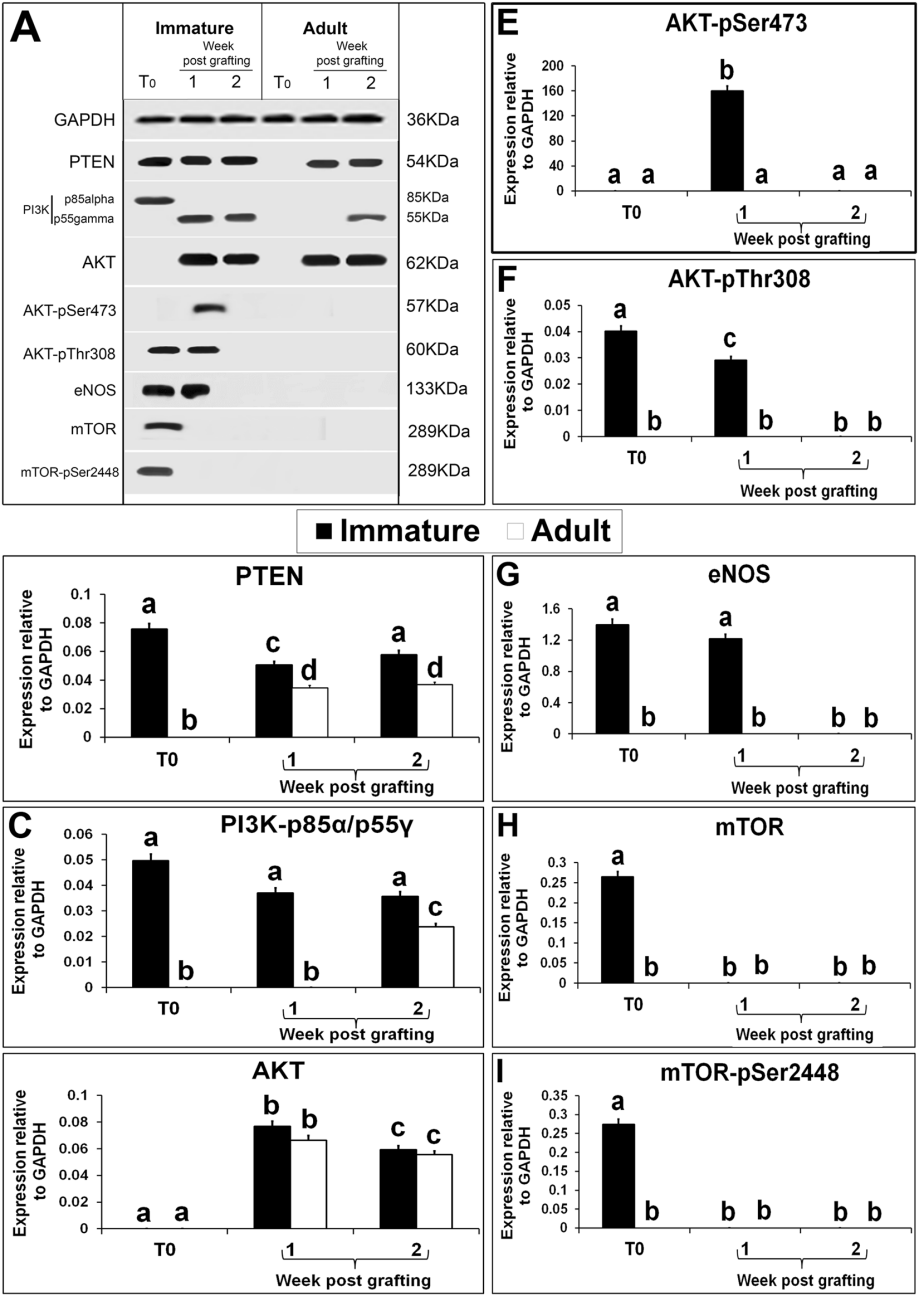
Western blot analysis of xenografted immature and adult testes at 1-and 2-wk-post grafting for expression of PI3K/AKT/mTOR pathway proteins. Protein expression in 6-day-old and 10-wk- old donor testes before grafting (T0) are presented as starting material. Representative blot (**A**) and densitometry analysis of (**B**) PTEN, (**C**) PI3Kp85alpha/gamma-pY467/199, (**D**) AKT, (**E**) AKT-pSer473, (**F**) AKT-pTyr308, (**G**) eNOS, (**H**) mTOR and (I) mTOR-pSer2448 protein. Y-axis represents intensity of bands relative to GAPDH. Data are presented as mean ± SEM. Bars with different letters are significantly different at P < 0.05.

The expression of PTEN proteins was present in immature testis but, was absent in adult testis (Fig. 6A). The expression level of PTEN in immature testis xenografts at 1 wk was lower than that in immature testis (Fig. 6B; P < 0.05) interestingly, its level increased in xenografts at 2 wk and was similar to that in immature testis (P > 0.05). The expression level of PTEN protein in adult testis xenograft at both collection time points was similar (P > 0.05) but, lower than that in xenografts from immature testis (P < 0.05). *PTEN* is a tumour suppressor gene and it regulates angiogenesis through both phosphatase-dependent and independent mechanisms^66^. The *PTEN* gene encodes a phosphatase that opposes the action of PI3K, thereby, reducing the level of activated AKT. AKT controls protein synthesis and cell growth by phosphorylation of mTOR^67^. Antibody against PI3K p85-alpha/p55 gamma (pY467/199) identified only alpha subunit (85KDa) in immature fresh testis but both forms of PI3K were absent from adult testis. Interestingly, gamma (55KDa) subunit of activated PI3K was present in xenografts of immature testis at both collection time points and in xenografts of adult testis at 2 wk only. It is reported previously that activated PI3K subunits, p55^γ^ and p85^α^ act in concert to mediate microtubule stabilization and rearrangement^68^. In this study, simultaneous expression of both the subunits was absent in the immature and adult testis as well as their xenografts. Expression of AKT, another subsequent downstream protein in PI3K pathway, was absent in both immature and adult testes. Nevertheless, the expression of AKT protein was present in xenografts of immature and adult testes at both the collection time points (Fig. 6D). The expression level of AKT protein was similar in xenografts of immature and adult testis collected at 1 wk (P > 0.05) but, it was reduced in xenografts collected at 2 wk (P < 0.05). To further confirm the role of AKT pathway in angiogenesis, we analysed the expression of the activated forms of AKT, p-AKT ser473 and p-AKT Thr308. Although expression of p-AKT Thr308 was present in immature testis, expression of p-AKT ser473 and p-AKT Thr308 was present in immature testis xenografts collected at 1 wk only. However, both forms of activated AKT were absent in xenografts of adult testis at both collection time points (Fig. 6E and F). These results show that AKT pathway is not activated in adult testis xenografts.

mTOR, a serine/threonine kinase is an important downstream target of PI3K/AKT pathway, which drives cell growth, proliferation and survival during angiogenesis^69^. Although the expression of mTOR and activated form of mTOR, mTORp2448, proteins was present in immature testis, it was absent in adult testis and in both immature and adult testis xenografts (Fig. 6H and I). These results rule out the role of mTOR in angiogenesis in testis xenografts. eNOS is another vital protein that regulates angiogenesis either by activation of PI3K/AKT pathway or through activation of growth factors such as ANGPT1. *In vivo* studies have shown that ANGPT1 induces angiogenesis through AKT phosphorylation and PI3K-mediated eNOS activation^70^. eNOS is a Ca^_^dependent protein involved in angiogenesis that is constitutively expressed in vascular endothelial cells and activated following exposure to VEGF^71^. In this study, eNOS protein expression was present in immature testis and its xenografts collected at 1 wk (Fig. 6A). The significant increase in VEGFR1 and eNOS level in immature testis xenografts at 1 wk, indicates their critical role in establishing angiogenesis in immature testis xenografts. The absence of expression of VEGFR1, VEGFR2, and eNOS could be the cause of poor angiogenesis in adult testis xenografts.

**Figure 7:**
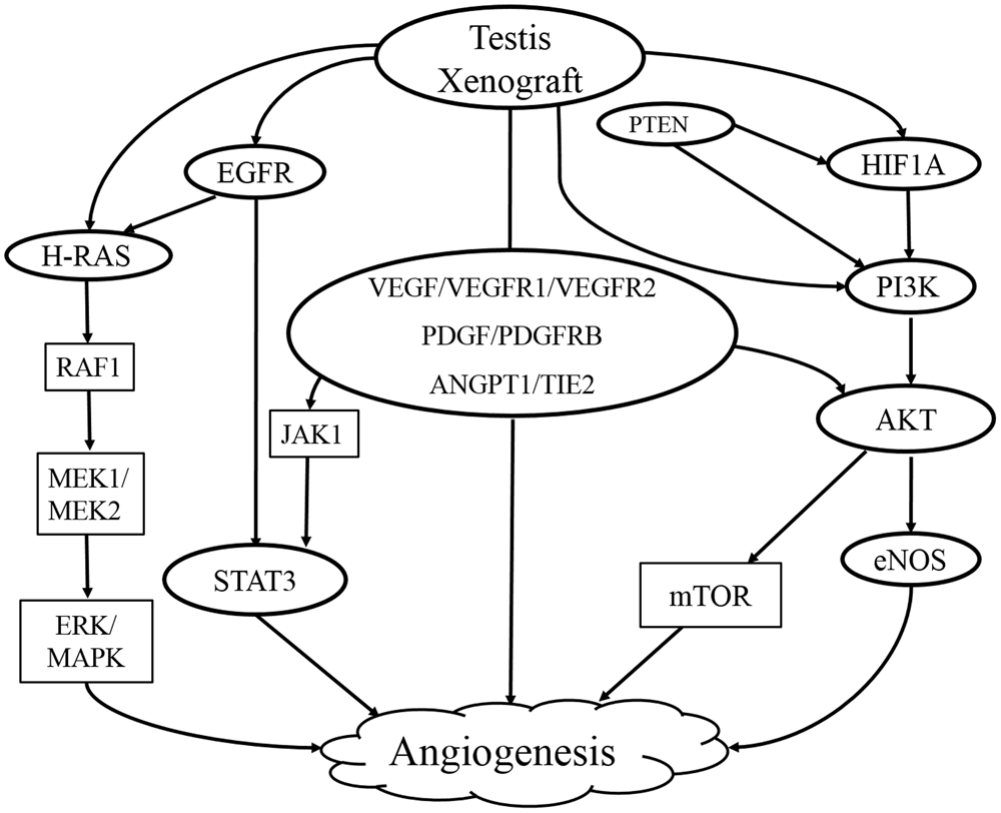
Schematic representation of pathways involved in angiogenesis of xenografted testis tissues. Signalling proteins denoted inside the oval shape were found to have role in testis xenograft angiogenesis and the ones indicated inside the rectangle shape did not.

To our knowledge, this is the first study that evaluated molecular mechanism responsible for angiogenesis in testis xenografts (Fig. 7). Xenografts from immature donor showed superior survival rate with the establishment of spermatogenesis and progression of undifferentiated germ cells into meiosis. This can be attributed to expression of several growth factors, receptors and activation of pathway proteins in immature testis and in its xenografts causing stimulation of angiogenesis. The absence or reduced expression of angiogenesis-specific proteins in adult testis and its xenografts could be the reasons for poor angiogenesis and the subsequent degeneration of xenografts. The angiogenesis in testis graft could be established either through eNOS activation via VEGF and PI3K/AKT or via EGFR- mediated STAT3 pathway. Though the expression of HIF1A and H-RAS proteins were present both in immature and adult xenografts, their downstream effector proteins (RAF/MEK/ERK/MAPK) were not activated, ruling out the role of ERK/MAPK pathway in angiogenesis. This study provides insight into angiogenesis mechanism that can be further utilized to augment testis xenografting efficiency.

## Material and Methods

### Animals and care

All animal procedures were performed in accordance with the relevant guidelines and regulations approved by the Institutional Animal Care and Use Committee (IACUC) of the Centre for Cellular and Molecular Biology (CCMB), Hyderabad, India (permit number 68/2015). Wistar rat (*Rattus norvegicus*) pups at age 6 days postpartum (refer to as immature) and at age 10 wk (refer to as adult) were used as testis donors. Immunodeficient male nude mice (nu/nu Balb/c) were kept under specific pathogen-free conditions, and food, water, and bedding were autoclaved before use. All the animals used were housed in 12 hours light: 12 hours darkness cycle at constant temperature and provided food and water *ad libitum*.

### Testis collection and xenografting

Testes were collected from immature (n = 44) and adult (n = 6) rats following euthanasia by CO_2_ inhalation. After collection, the epididymis was removed and testis was weighed. The tunica albuginea from immature rat testis was removed before grafting. The average weight of immature testis was ~7 mg at the time of grafting. For adult testis, after removal of tunica albuginea, the parenchymal tissue was cut into small pieces (~7 mg). The testis pieces were placed on ice in Dulbecco’s modified Eagle’s medium/Ham’s F12 (DMEM/F12) HEPES (Gibco; Invitrogen, Carlsbad, CA, USA) until grafting. Four rat testes (immature) or 4 pieces of testis (adult) were ectopically grafted onto the back of castrated nude mice (nu/nu Balb/c, 6-8-wk-old, n = 44). Briefly, mice were anesthetized with ketamine (0.1 mg/kg body weight (BW) and xylazine (0.5 mg/kg BW) in sterile physiological saline. For castration of recipient mice, a ventral medial incision was given in the abdomen and the testes were removed following which the peritoneum and skin sutured closed using absorbable suture (Ethicon; Somerville, NJ, USA). During the same surgery, each mouse received two incisions (~5 mm) on each side of the back (total four incisions). One piece of testis was inserted through each incision. The incisions were sutured closed and the mice were allowed to recover and returned to their cages.

### Recipient mice analysis, recovery and histological analysis of xenografts

For the collection of xenografts and seminal vesicles, recipient mice were euthanized by CO_2_ inhalation. The fresh testis from immature and adult rats served as starting material (T0 from here onwards; n = 6 for each). Xenografts were collected from mice at 1 and 2 wk post grafting to observe the expression of angiogenesis signalling-specific proteins by western blot analysis (n = 6 for each time point). At 8-wk-post grafting, the xenografts were recovered, weighed and taken for histological analysis. The weight of the seminal vesicle from recipient mice was measured as an indicator of bioactive testosterone production by the grafted tissue^1, 4, 7^. Average weight of seminal vesicle from non-grafted intact adult males (nu/nu Balb/c, 6–8-wk old, n = 8) was taken as control. The percentage of recovered xenografts was calculated for mice in which xenografts were found. It was also recorded when no xenografts were recovered from a mouse. For histological analysis, xenografts and control testis tissues were fixed in Bouin’s solution followed by tissue dehydration with increased concentrations of alcohol in a series. After processing, the tissues were embedded in paraffin, sectioned (7 µm thick), stained with haematoxylin and eosin (H&E), dehydrated, mounted in Vectamount (Vector Laboratories; Burlingame, CA, USA) and observed under a light microscope (Nikon; Melville, NY, USA) for morphological evaluation. Establishment of spermatogenesis was assessed by morphological evaluation as previously described^72^. In xenografts where seminiferous tubules were observed, all tubules were analysed and classified either as degenerated or non-degenerated. A graft was classified as degenerated which did not contain distinct cell types and no tubules were found and, non-degenerated if it contained even a single seminiferous tubule with spermatogenesis. The percentage of seminiferous tubules and degenerated tubules within a graft was also calculated. Spermatogenesis was assessed by examining the most advanced germ cell type in the recovered xenografts.

### PCNA immunostaining of donor testis and xenograft

The tissue sections from xenografts collected at 8 wk post grafting were stained with proliferating cell nuclear antigen (PCNA) antibody to evaluate cell proliferation in seminiferous tubules. The fresh testis from immature and adult rats served as control (T0). Dilutions of primary and secondary antibodies were done in PBS with 1% BSA (BSA; Sigma). Briefly, after deparaffinization and rehydration, sections were blocked with 10% FBS and 3% BSA in PBS for 30 minutes, incubated with mouse anti-PCNA antibody (1:200; Millipore) overnight at 4 °C, washed several times with PBS, incubated with 3% H_2_O_2_ for 10 min, washed three times with PBS, incubated with rabbit anti-mouse horseradish peroxidise conjugated secondary antibody (1:200; Calbiochem) for 30 min at 37 °C, rinsed three times with PBS, incubated for 3 to 5 min using a DAB Substrate Kit (Vector Laboratories) according to the manufacturer’s instruction, rinsed thoroughly in distilled water, and mounted. Negative control sections were incubated in mouse isotype control antibodies (Santa Cruz Biotechnology, Inc.) instead of primary antibodies at the same concentration, but all other procedures remained the same. PCNA-stained tubule cross sections were scored for the presence of PCNA-positive germ cells and Sertoli cells (distinguished by typical nuclear morphology and location in the seminiferous tubules) in each section. An average of 50–100 tubules/cross-section and 15–30 random fields at 400x magnification per recipient mouse were examined.

### Western blotting analysis of donor testes and xenografts

Total protein from xenografts collected 1 and 2 wk post grafting was extracted upon homogenization by sonication in a dissolving buffer (7 M urea, 2 M thiourea, 4% CHAPS, 18 mMTris–HCl, 14 mMTris– Base, 0.2% Triton-X and 50 mM dithiothreitol). Single-strength ProteCEASE-50, EDTA-free protease inhibitor (G-Biosciences, St. Louis, MO, USA) was added to the dissolving buffer before protein extraction. Protein from testis of immature and adult rats served as a representation of starting material (T0). The lysed samples (40 µg) were subjected to electrophoresis on 12% SDS–polyacrylamide gel. The gels were transferred onto PVDF membranes (Millipore; Billerica, MA, USA). The membranes were blocked with Starting Block TBS blocking buffer (Thermo Scientific; Waltham, MA, USA) for 1 h at room temperature. The blocked membranes were incubated with one of the following primary antibodies for overnight at 4 °C: hypoxia inducible factor1 alpha (HIF-1A, 1:1000), epidermal growth factor receptor (EGFR, 1:1000), angiopoietin receptor TIE2 (TIE2, 1:1000), mitogen-activated protein kinase (MAPK/ERK,1:500), phospho-p44/42 MAPK (ERK1/2-Thr202/Tyr204, 1:500), serine-threonine protein kinase AKT (AKT, 1:1000), endothelial NOS (eNOS, 1:1000), glyceraldehyde-3-phosphate dehydrogenase (GAPDH; 1:1000) (all from Thermo Scientific) vascular endothelial growth factor receptor 1 (VEGFR1, 1:1000); Angiopoietin 1 (ANGPT1, 1:500) (both from Santa Cruz Biotechnology; Dallas, TX, USA); vascular endothelial growth factor (VEGF, 1:100), vascular endothelial growth factor receptor 2 (VEGFR2, 1:500), platelet-derived growth factor (PDGF, 1:100), platelet-derived growth factor receptor beta (PDGFRB, 1:100), AKT-pThr308 (1:100), AKT-pSer473 (1:500), PI3K-p85 alpha/p55 gamma(pY467/199) 1:500, mammalian target of rapamycin (mTOR, 1:500), mammalian target of rapamycin pSer2448 (mTOR-pSer2448, 1:100), phosphatase and tensin homolog (PTEN, 1:500), serine/threonine-protein kinase c-RAF (RAF1,1:500), mitogen-activated protein kinase kinase 1/2 (MEK1/MEK2, 1:500), signal transducer and activator of transcription 3 (STAT3, 1:100), STAT3-pY705 (1:100), janus kinase 1 (JAK1, 1:100; all from One World Lab; San Diego, CA, USA). The membranes were then washed with TBS-T and incubated with goat anti-rabbit, goat anti-mouse or rabbit anti-goat HRP-conjugated secondary antibody (1:10000; both from Thermo Scientific) in TBS-T for 1 h at room temperature. After washing with TBS-T, immunoreactivity was detected by chemoluminescence using a C-DiGit Blot Scanner (LI-COR Biosciences; Lincoln, NE, USA) against SuperSignal West Femto chemiluminescent substrate (Thermo Scientific), and the generated signal was analysed using a densitometer. To control protein loading on the gels, the membranes were further probed with GAPDH antibody.

### Statistical analyses

The results were presented as mean ± S.E.M. The statistical analyses were performed by ANOVA. Significant differences between the means were determined by analysing the data using the Tukey honest significance difference (Tukey HSD) test. The level of significance was set at P < 0.05.
